# Prediction of Coronary Artery Disease Extent and Severity Using Pulse Wave Velocity

**DOI:** 10.1371/journal.pone.0168598

**Published:** 2016-12-22

**Authors:** Joseph Chiha, Paul Mitchell, Bamini Gopinath, George Burlutsky, Adam Plant, Pramesh Kovoor, Aravinda Thiagalingam

**Affiliations:** 1 Centre for Heart Research, Westmead Millennium Institute, University of Sydney, Sydney, New South Wales, Australia; 2 Centre for Vision Research, Department of Ophthalmology, Westmead Millennium Institute, University of Sydney, Sydney, New South Wales, Australia; Nagoya University, JAPAN

## Abstract

**Background:**

Pulse-wave velocity (PWV) measures aortic stiffness. It is an independent predictor of cardiovascular events and mortality, yet there is paucity in the literature on its association with the severity and extent of coronary artery disease (CAD).

**Methods:**

To examine the utility of PWV in predicting CAD burden in men and women the PWV was determined in 344 patients (Men = 266, Women = 78) presenting for invasive coronary angiography for the assessment of suspected CAD. Pearson correlations and multivariate analysis were used to evaluate the relationship between these coronary scores, PWV and traditional cardiovascular risk factors.

**Results:**

Compared to men, women with chest pain had lower mean Extent scores (19.2 vs. 35.6; p = 0.0001) and Gensini scores (23.6 vs. 41.9; p = 0.0001). PWV was similar between men and women (12.35 ± 3.74 vs. 12.43 ± 4.58; p = 0.88) and correlated with Extent score (r = 0.21, p = 0.0001) but not Gensini or vessel score (r = 0.03, p = 0.64 and r = 0.06, p = 0.26, respectively). PWV was associated with Extent score in men (B = 2.25 ± 0.78, p = 0.004 for men and B = 1.50 ± 0.88, p = 0.09 for women). It was not a predictor of Gensini score (B = -0.10, P = 0.90).

**Conclusion:**

PWV correlates with the extent of CAD, as measured by the ‘Extent’ score in men more than women. However, it does not correlate with the severity of obstructive CAD in either gender.

## Introduction

The diagnosis of coronary artery disease (CAD) involves risk factor assessment, combined with stress testing and imaging to select patients for coronary angiography. It is known that the incidence of coronary artery disease in people presenting with chest pain is dramatically different between men and women with significant CAD absent in up to 60% of women and 30% of men. [1–3] Further, angiographically normal coronary arteries are present in approximately 20–30% of patients with suspected angina. [2, 4] Patients with normal coronary arteries have a favorable long-term prognosis with similar mortality and coronary morbidity to that of the general population and substantially better than for those with minor coronary irregularities or obstructive atherosclerotic lesions. [2, 5, 6]

Carotid—femoral pulse wave velocity (PWV) is currently considered the current gold-standard method for assessing arterial stiffness. [7, 8] It is regarded as an independent predictor of risk of cardiovascular events and mortality and is endorsed by the European Society of Hypertension in the workup of hypertension and assessment of its severity. [8–10] Despite this association, there is paucity in the literature on the utility of PWV for the prediction of the stenosis severity and extent of CAD. The study aimed to assess the correlation between aortic stiffness as measured by PWV and the extent and severity of CAD as assessed by coronary angiography in men and women.

## Methods

### Study Population

1680 participants were recruited for the AHES from January 2010 and January 2012. These were people who presented to Westmead Hospital, Sydney, Australia for invasive coronary angiography for the assessment of chest pain due to suspected CAD. Patients were referred for investigation by a cardiologist for outpatient or inpatient investigation. The decision to pursue angiography was made by the referrer who was not involved in the subsequent recruitment of the patients to the study. Participants were consented to the study prior to or following invasive coronary angiography. Exclusion criteria were presentation with acute myocardial infarction, unstable angina, cardiogenic shock. Of the 1680 examined in the AHES study, a total of 489 participants were excluded because they had a previous history of coronary artery bypass grafting (n = 191) and/or previous coronary artery stent (n = 298). Of the remaining 848 in the study, 343 agreed to have carotid-femoral PWV assessments performed as part of the study (Men = 266, Women = 78).

The protocol was approved by the Westmead Hospital (Sydney West Local Health Network) ethics committee, and was performed in accordance with the Declaration of Helsinki. All patients provided written informed consent.

### Measurement of Pulse-Wave Velocity

Aortic stiffness was assessed with carotid-femoral pulse-wave analysis using applanation tonometry (SphygmoCor, Atcor Medical, Sydney, Australia) that allowed on-line pulse wave recording and automatic calculation of PWV. Measurement was repeated over 10 different cardiac cycles, and the mean value was used for the final analysis. [11–14] The carotid-femoral path length was measured directly with a tape measure (D). The tonometer was applied to the right carotid and then right femoral artery to obtain pulse-wave travel time (t). Carotid-femoral PWV was calculated as PWV = 0·80D/t (m/s) as proposed recently. [15]

### Evaluation of Coronary Artery Disease

Diagnostic coronary angiography was performed via a femoral or radial approach. Selective coronary injections were filmed in standard projections with a Siemens Bi-Plane radiographic unit (Siemens Healthcare, Germany). Cine runs were stored at the time of acquisition in DICOM format.

All angiograms were analyzed offline by a cardiologist (author J.C.) blinded to the medical history and adjunctive investigations. Two orthogonal views were examined in end-diastole and the image with the most severe stenosis was used. Each lesion that was visually scored as greater than 50% luminal obstruction in a vessel that was ≥1.5mm diameter was further analyzed by quantitative coronary analysis (QCA) using validated computerized edge-detection software (QCAPLUS, Sanders data Systems, Palo Alto, California, USA) [16]. Coronary angiograms were scored using three methods:

1. *Vessel score*: was calculated based on the number of vessels with significant obstructive coronary disease defined as greater than 50% stenosis. [17] This definition was used for the left main coronary artery, right coronary, left anterior descending and left circumflex arteries. Scores ranged from 0 to 4, depending on the number of vessels with greater than 50% stenosis. [18] Left main artery stenosis was scored as double vessel disease.
2. *Gensini Score* [19]: This divides the three coronary arteries into several sub-segments. The percent diameter stenosis is scored from zero to 32 depending on the severity of the stenosis. Each segment is given a multiplying factor (from 0.5 for the distal segment to 5 for the left main coronary artery) depending on the significance of the myocardial area supplied by that segment. The sum of the scores gives the Gensini score, which provides an indication of the severity of coronary artery disease stenoses and has been used as a tool to assess the relationship between coronary and other vascular disease. [19–22]
3. *Extent Score*: First published by Sullivan et al. [23] this indicates the percentage of the coronary arterial tree involved by angiographically detectable coronary atheroma independent of the stenosis severity. The proportion of the vessel with irregularity is multiplied by a factor for each vessel representing the length of the artery. The scores for each vessel were added to give a total score out of 100, that is the percentage of the coronary intimal surface area containing coronary atheroma

### Statistical Analysis

Data were analyzed using SAS 9.2 (Statistical Analysis Package, SAS Institute, 2011, North Carolina, USA). All categorical data were reported as percentages and continuous variable expressed as mean ± standard deviation (SD). Student’s *t*-test was used for the comparison of demographic data that were continuous variables and chi-squared analysis for categorical variables. Pulse-wave velocity was adjusted for waist:height ratio and systolic blood pressure. Relationships between PWV, gender, age, cardiovascular risk factors and the coronary artery scores were assessed using Pearson correlations. Risk factors were analyzed as binary variables for the correlation models. The effect of each independent clinical variable on the Extent and Gensini scores were examined using multiple regression models both as a grouped population as well as divided into gender. Data is provided as unstandardized coefficients (B) with corresponding p-values. PWV results were further dichotomized into <10 meters/second and ≥10 meters/second as recommended [24] and t-tests were performed. Statistical significance was present when p < 0.05. S1 and S2 Tables provide raw data and variables analyzed.

## Results

### Patient Characteristics

The study cohort was predominantly men (77%) with an average age of 59.4 years and Caucasian background (Table 1). There was no significant difference in the presence of traditional cardiac risk factors except for a higher proportion of women having hypertension (71.8 vs. 56.6%, p = 0.02). Both the Gensini and Extent score were substantially higher for the male cohort compared to women (p = 0.0002 and p < 0.001 respectively). The average PWV was similar between men and women (12.35 m/s vs. 12.43 m/s respectively, p = 0.88).

**Table 1:** Demographic and clinical characteristics of participants stratified by gender.

Characteristics	Women (n = 78)	Men (n = 266)	p-value
Sex (%)	23	77	<0.001
Age, *yrs*	63.6 (11.0)	59.4 (11.5)	<0.001
Body Height, m	1.60 (0.1)	1.72 (0.08)	<0.001
Body Weight, kg	70.4 (12.3)	82.6 (14.3)	<0.001
Body mass index, kg/m^2^	27.3 (4.3)	28.0 (4.1)	0.21
Waist:height ratio	57.1 (7.2)	57.2 (6.6)	0.89
Blood Pressure (mmHg)			
Systolic	126.6 (20.5)	128.3 (18.3)	0.49
Diastolic	69.2 (12.1)	73.5 (11.2)	0.005
Mean arterial	88.4 (12.7)	91.7 (12.1)	0.03
Ever Smoked (%)	25.6	26.7	0.85
History of Hypercholesterolemia (%)	81.9	78.2	0.49
History of hypertension (%)	71.8	56.6	0.02
History of diabetes	30.8	32.1	0.83
Current medications (%)			
Aspirin	37.2	39.9	0.67
Clopidogrel/Prasugrel/Ticagrelor	12.8	13.2	0.94
Nitrate	5.1	4.1	0.71
Beta-blocker	17.9	21.4	0.50
Calcium Channel Blocker	28.2	12.8	0.001
ACE-inhibitor	20.5	14.7	0.22
Angiotensin II Receptor antagonist	28.2	16.9	0.03
alpha-blocker	3.9	3.4	0.84
Statin	53.9	38.7	0.02
Other antianginal agent	6.4	1.1	0.007
Pulse-wave velocity (m/s)	12.43 (4.58)	12.35 (3.74)	0.88
Gensini Score	23.6 (34.6)	41.9 (39.0)	<0.001
Extent Score	19.2 (22.5)	35.6 (30.7)	<0.001
Vessel Score	0.8 (0.9)	1.3 (1.1)	<0.001

Continuous variables are expressed as mean (±SD) and categorical data as counts; n (%). m/s, meters/second; p-value significant at p < 0.05.

Gender was the most significant factor for the Gensini score (r = 0.20, p = < 0.001), followed by smoking (r = 0.11, P = 0.04). PWV was not associated with Gensini score for this cohort (r = 0.03, p = 0.61). By contrast, several risk factors were positively correlated with Extent score. Sex was the most correlated (r = 0.23, p < 0.001), followed by PWV (r = 0.21, p < 0.001), diabetes (r = 0.15, p = 0.005) and age (r = 0.13, p = 0.01). PWV was not correlated with the Vessel score (r = 0.06, p = 0.26).

Multiple regression analyses revealed that gender was the most significant risk factor for both the Gensini and Extent score. When examining the factors by gender, only diabetes and PWV were significantly associated with Extent score in men (B = 12.25, p = 0.004 and B = 2.25, p = 0.004 respectively). However, this association was not significant in women. The vessel score was not associated with PWV in either the grouped cohort or when examined by gender.

**Fig 1:**
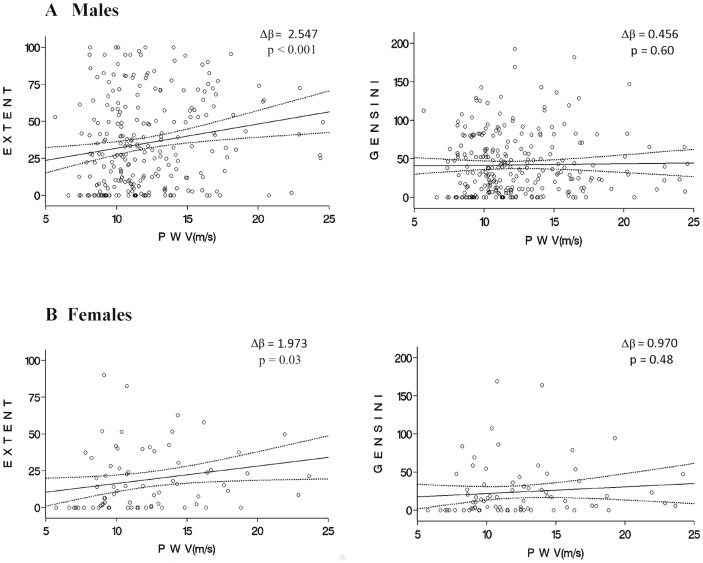
The relationship of pulse-wave velocity (PWV) with Extent and Gensini score in men (A) and women (B) using a univariate linear regression analysis. *Caption*: Solid line = regression line, dotted line = 95% confidence interval. The Δβ represents the change in value of the Extent or Gensini score with every 1 m/s increase in the PWV result. Significant dependent correlations have p < 0.05. PWV, pulse-wave velocity.

Tables 2 and 3 summarize the Extent and Gensini scores in men and women when PWV was dichotomized to <10 m/s and ≥10 m/s. There was no significant difference between the two PWV groups when examining the Gensini score in any group. However, for the Extent score, a PWV <10m/s was associated with a significantly lower score for the grouped cohort (24.8 vs. 34.8, p = 0.006). The difference in Extent score for men was very near significant (29.4 vs. 37.9, p = 0.05) however for women there was no difference (13.7 vs. 22.5, p = 0.10). Although the dichotomizing of PWV suggests the presence of at least one vessel with significant obstruction (p = 0.03 for the grouped cohort), it was not able to differentiate between 1, 2 or 3 vessel disease (p = 0.19 for the grouped cohort). There was a positive relationship between PWV and Extent score for men and women, with the relationship in men being more pronounced (Fig 1).

**Table 2:** Average Extent score for men and women with Pulse-wave velocity <10 m/s and ≥10 m/s.

	Grouped (n = 343)			Male (n = 266)			Female (n = 78)		
PWV	n	Extent score (LCL,UCL)	p	n	Extent score (LCL,UCL)	p	n	Extent score (LCL,UCL)	p
**< 10 m/s**	98	24.8 (18.9, 30.8)		71	29.4 (21.9, 39.4)		28	13.7 (5.5, 21.9)	
			0.006			0.05			0.10
**≥10 m/s**	245	34.8 (31.0, 38.6)		195	37.9 (33.5, 42.2)		50	22.5 (15.8, 29.2)	

PWV, Pulse-wave velocity; m/s, meters/second; LCL, lower confidence limit; UCL, upper confidence limit; p-values < 0.05 are significant

**Table 3:** Average Gensini score for men and women with Pulse-wave velocity <10 m/s and ≥10 m/s.

	Grouped (n = 343)			Male (n = 266)			Female (n = 78)		
PWV	n	Gensini score (LCL,UCL)	p	n	Gensini score (LCL,UCL)	p	n	Gensini score (LCL,UCL)	p
**< 10 m/s**	98	36.1 (28.3, 43.9)		71	43.8 (34.0, 53.6)		28	16.8 (7.4, 26.1)	
			0.61			0.64			0.14
**≥10 m/s**	245	38.5 (33.6, 43.3)		195	41.3 (35.9, 46.7)		50	27.5 (16.4, 38.5)	

PWV, Pulse-wave velocity; m/s, meters/second; LCL, lower confidence limit; UCL, upper confidence limit; p-values < 0.05 are significant

## Discussion

In a cohort of people with suspected ischemic chest pain referred for coronary angiography, we found an independent association between carotid-femoral PWV and atheroma burden and showed that PWV correlated with the extent of coronary atheroma but not with stenosis severity as assessed with Gensini score in either gender. To the best of our knowledge, this is the first study to examine potential gender differences in using PWV to predict angiographic coronary artery disease.

Our study used the Extent score to determine the burden of coronary atherosclerosis in a large population presenting for the evaluation of suspected angina. The Extent score provided anatomical perspective on the diffusion of atherosclerosis independent of stenosis severity reflected by the Gensini score. [19, 23] It has been shown previously by Bigi et al. [25] that vascular risk factors were associated with the extent of CAD than the number of vessels affected by significant obstructive stenoses. Further, the pattern of CAD in women substantially differed to that in men necessitating non-traditional methods of reporting CAD. By contrast, the Gensini score is a measure of the severity of coronary stenoses graded in proportion to the significance of their location. [19] It is well correlated with the Extent score. [26, 27] However, there is poor correlation between the occurrence and location of a myocardial infarction and the angiographic severity of luminal narrowing. [28] This is because the site of myocardial infarction occurs predominantly by occlusive thrombus or plaque rupture at sites with a non-obstructive coronary stenosis. [29]

In our study, only the Extent score was correlated significantly with age and the presence of diabetes. It is known that diffuse coronary atherosclerosis increases with age and is also significantly more prevalent in men. [30] Further, obstructive CAD is more prevalent in symptomatic men than women such that up to 60% of women and 30% of men who present with angina have either normal arteries or non-obstructive lesions. [3, 31] It is likely that the Extent score represents the chronic, progressive component of atherosclerosis and the Gensini score predominantly correlates with gender as obstructive CAD is more pronounced in men.

Pulse-wave velocity has been shown in a recent meta-analysis to predict future cardiovascular events and risk stratification, independent of the presence of other risk factors. [10] PWV may be a surrogate endpoint representative of the long-term influence of both the established and unknown vascular risk factors. [7] In keeping with other studies, we found no significant difference in the PWV between men and women. [10, 24] Of note the average PWV was above the 10m/s suggested previously [24] and may reflect the different population in our study. Published PWV guidelines represent a population without the presence of coronary artery disease, angina or risk factors aside from hypertension. It is therefore not unexpected that the average PWV is higher in our study cohort.

Our study suggests that PWV can be used to predict the extent of coronary atheroma but not stenosis severity. Interestingly, PWV was not a predictor of Gensini score in women. The borderline lack of association of Extent score with PWV in women probably reflects their lower number in the study and therefore may have underestimated the overall association as illustrated by the significant association in the grouped cohort.

The study by Coutinho et al. [32] found that PWV was independently associated with the presence and quantity of coronary artery calcification in treated hypertensive individuals. Kullo et al. [33] further demonstrated this association in a community-based cohort with no history of myocardial infarction. The presence of coronary artery calcification is representative of total plaque burden along the vessel. [34] As such, coronary calcification can be regarded as a reliable correlate of total coronary atherosclerosis. In contrast to our study, both these studies were in a population of asymptomatic individuals.

Women with suspected ischemic chest pain undergoing invasive coronary angiography have less extensive epicardial atheroma, also presenting with less obstructive epicardial stenoses and often with microvascular dysfunction. [35] Our results support this with the lower Extent and Gensini scores for women compared to men. Women can display evidence of ischemia on functional assessment (such as pressure wire studies, myocardial perfusion imaging and magnetic resonance imaging) without obstructive epicardial coronary disease, often unrelated to the presence of risk factors. [36, 37] Chest pain in women is likely to have a different pathophysiological mechanism compared to men and traditional risk factors may play an alternative role. It is not surprising therefore, that arterial stiffness as measured by carotid-femoral PWV is possibly less reliable at predicting diffuse coronary atheroma in the female subgroup.

## Conclusion

PWV may be associated with diffuse atheroma. This positive correlation between PWV and Extent score suggests that PWV may be predictive of future cardiovascular events due to the established link between Extent score and cardiovascular risk. Although aortic stiffness is an independent marker for future cardiovascular risk, our study shows the carotid-femoral PWV cannot predict the underlying obstructive coronary disease in symptomatic individuals presenting for the investigation of suspected ischemia.

### Limitations

Our study represents a group of patients from a single center with a broad demographic, all of who were symptomatic and being investigated and medically treated for suspected ischemia. This limits the generalizability of our results to similar care settings and the general population. Given the numbers of people who were not included in the analysis due to having exclusion criteria or inadequate coronary angiograms and reluctance to consent to having the PWV assessment done, there is some selection bias. The low proportion of women recruited in the study could have influenced the outcomes. Importantly, the association of PWV with Extent scores in women may be under-estimated. This does highlight the lower number of women presenting for coronary angiography as well as the perceived lower risk of ischemic heart disease. Selection bias cannot be completely excluded even after adjusting for a wide range of patient characteristics. Last, we did not collect information on microvascular or endothelial dysfunction or long-term cardiovascular outcomes.

## Supporting Information

S1 Table Variables. (DOCX)

S2 Table Data. (XLSX)
