# Supplementary material for: Prediction of Coronary Artery Disease Extent and Severity Using Pulse Wave Velocity
Source: PLoS One. 2016 Dec 22;11(12):e0168598. doi: 10.1371/journal.pone.0168598 (PMC5179020; doi:10.1371/journal.pone.0168598)
Supplement: S1 Table — (DOCX) [file pone.0168598.s001.docx]

| Variables in Creation Order | | |
| --- | --- | --- |
| # | Variable | Label |
| 1 | IDNUM | ID |
| 2 | Age | Age |
| 3 | Sex | 1-Female, 2-Male |
| 4 | Extent | Extent Score |
| 5 | Gensini | Gensini Score |
| 6 | Height3 | Height (m) |
| 7 | Weight3 | Weight (kg) |
| 8 | BMI | Body Mass Index (kg/m**2) |
| 9 | WHR | Waist/Height Ratio |
| 10 | Systbp3 | Systolic BP |
| 11 | Diasbp3 | Diastolic BP |
| 12 | MAP | Mean Arterial |
| 13 | Smoker2 | Ever Soked(%) |
| 14 | Hypert | History of Hypertension(%) |
| 15 | Lipid | History of Hypercholesterolemia (%) |
| 16 | Diabet | History of Diabetes (%) |
| 17 | nNmVs | Vessel Score |
| 18 | BB | Beta-Blocker (%) |
| 19 | CCB | Calcium Channel Blocker (%) |
| 20 | Nitrate | Nitrate (%) |
| 21 | PWV | Pulse-wave Velocity (m/s) |
| 22 | Aspirin | Aspirin (%) |
| 23 | Clorid | Clopidogrel/Praugrel/Ticagrelor (%) |
| 24 | OthAntiAng | Other Antianginal Agent (%) |
| 25 | ACE | ACE-inhibitor (%) |
| 26 | ARB | Angiotensin II Receptor Antagonist (%) |
| 27 | A_Bloker | Alfa-Bloker (%) |
| 28 | Statin | Statin (%) |
